# Polymer biodegradation by *Halanaerobium* promotes reservoir souring during hydraulic fracturing

**DOI:** 10.1128/aem.02253-24

**Published:** 2025-04-09

**Authors:** Gabrielle Scheffer, Anirban Chakraborty, Kaela K. Amundson, Rohan Khan, Michael J. Wilkins, Paul Evans, Casey R. J. Hubert

**Affiliations:** 1 Geomicrobiology Group, Department of Biological Sciences, University of Calgary 98634, Calgary, Alberta, Canada; 2 Department of Biological Sciences, Idaho State University 166927, Pocatello, Idaho, USA; 3 Department of Soil and Crop Sciences, Colorado State University 124498 , Fort Collins, Colorado, USA; 4 Chevron Technical Center, Houston, Texas, USA; University of Illinois Urbana-Champaign, Urbana, Illinois, USA

**Keywords:** *Halanaerobium*, sulfidogenesis, produced water storage, hydraulic fracturing, guar gum, fermentation, souring

## Abstract

Hydraulically fractured shale reservoirs have facilitated studies of unexplored niches in the continental deep biosphere. In high-salinity North American shale systems, members of the genus *Halanaerobium* seem to be ubiquitous. Polymers like guar gum used as gelling agents in hydraulic fracturing fluids are known to be fermentable substrates, but metabolic pathways encoding these processes have not been characterized. To explore this, produced water samples from the Permian Basin were incubated both at 30°C to simulate above-ground storage conditions and at 60°C to simulate subsurface reservoir conditions. Guar metabolism coincided with *Halanaerobium* growing only at 30°C, revealing genes for polymer biodegradation through the mixed-acid fermentation pathway in different metagenome-assembled genomes (MAGs). Whereas thiosulfate reduction to sulfide is often invoked to explain the dominance of *Halanaerobium* in these settings, genes for thiosulfate metabolism were lacking in *Halanaerobium* genomes with high estimated completeness. Sulfide production was observed in 60°C incubations, with corresponding enrichment of *Desulfohalobium* and *Desulfovibrionaceae* that possess complete pathways for coupling mannose and acetate oxidation to sulfate reduction. These findings outline how production of fermentation intermediates (mannose and acetate) by *Halanaerobium* in topside settings can result in reservoir souring when these metabolites are introduced into the subsurface through produced water reuse.

**IMPORTANCE:**

Hydraulically fractured shale oil reservoirs are ideal for studying extremophiles such as thermohalophiles. During hydraulic fracturing, reservoir production water is stored in surface ponds prior to reuse. Microorganisms in these systems therefore need to withstand various environmental changes such as the swing between warm downhole oil reservoir temperatures and cooler surface conditions. While most studies on hydraulically fractured oil reservoirs mimic the environmental conditions found in oil wells, this study follows this water cycle during fracking and the associated microbial metabolic potential during topside-produced water storage and subsurface oil reservoir conditions. Of particular interest are members of the genus *Halanaerobium* that have been reported to reduce thiosulfate contributing to souring of oil reservoirs. Here, we show that some *Halanaerobium* strains were unable to grow at hotter temperatures reflective of oil reservoir conditions and lack genes for thiosulfate reduction, despite the proposed importance of this metabolism in other studies. Rather, it is likely that these organisms metabolize complex organics in fracking fluids at lower temperatures, thereby generating substrates that support reservoir souring by thermophilic sulfate-reducing bacteria at higher temperatures. In this way, *Halanaerobium* promotes souring indirectly by feeding sulfate-reducing microorganisms fermentation products (e.g., acetate and hydrogen) rather than via direct sulfidogenesis via thiosulfate reduction. Therefore, the novelty of this research is not within the detection of known oil reservoir colonizing bacteria but rather in the relationship between bacteria and the indirect involvement of *Halanaerobium*, promoting souring throughout the produced water reuse cycle.

## INTRODUCTION

Studies of the terrestrial deep biosphere have advanced in recent years due to hydraulic fracturing for oil and gas production (1–3), showcasing polyextremophiles able to contend with high temperatures and salinities (4–9). Shale formations ranging from 45°C to 190°C (10) with salinities of 40,000–280,000 mg/L (11) are further impacted by organic polymers that are used in the hydraulic fracturing process. Common organic polymers used as gelling agents such as guar gum, carboxymethyl cellulose, or polyacrylamide-based compounds have all been shown to be biodegradable as fermentable substrates (for guar gum and carboxymethyl cellulose) or sources of nitrogen (for polyacrylamide-based polymers; 12–15).

Among the most commonly detected microorganisms in hydraulically fractured shale reservoirs are members of the genus *Halanaerobium*. These bacteria appear to be ubiquitous throughout most North American shale reservoir operations, including the Marcellus, Barnett, Antrim, and Haynesville systems (2). *Halanaerobium* spp. are known to ferment a wide range of carbohydrates, including guar gum (5, 12), but can also reduce elemental sulfur or thiosulfate to generate sulfide (6). Formation of precursors to sulfide biogenesis (e.g., zero-valent polysulfide or thiosulfate intermediates) depends on temperature, oxygen, pH, metals, and the presence of other sulfur species (13). While some studies have measured very low to non-detectable levels of thiosulfate in shale reservoir systems (5, 6, 14, 15), others have amended sulfur species as electron acceptors at artificially high levels to provoke sulfide generation in incubations (16, 17). While the most well-understood paradigm for oil reservoir souring involves sulfate reduction, knowledge gaps persist regarding the mechanisms of souring in shale systems, where it is popular to invoke reduction of thiosulfate or elemental sulfur by *Halanaerobium* spp. *in situ* (13, 16, 17). These proposals are supported by the physiology of pure cultures, including the type strain *Halanaerobium congolense* (18).

Limited water sources in the vicinity of hydraulic fracturing operations lead many companies to reuse produced water from subsurface reservoirs to generate subsequent batches of fracturing fluids (19). As part of this process, microbial populations are exposed to different temperatures for prolonged periods between produced water stored in what are termed storage ponds at the surface and reintroduction into the reservoir. In the Permian Basin, shale formations tend to be around 60°C, while surface storage ponds are commonly around 30°C (20, 21). Little is known about the persistence and activity of microorganisms in topside storage ponds, the populations that can survive for prolonged periods, the type of metabolic ability they have, and whether that metabolism influences operations. Given that *Halanaerobium* strains have only been isolated below 42°C, it is not well understood how they may withstand the temperature fluctuations between hotter subsurface reservoirs and cooler topside storage ponds (16, 17). The objectives of this study were to better understand how the genomic and biogeochemical dynamics between produced water storage ponds and hydraulically fractured oil reservoir conditions can contribute to carbon and sulfur cycling.

## RESULTS

### Metabolic processes at 30°C and 60°C

**Fig 1:**
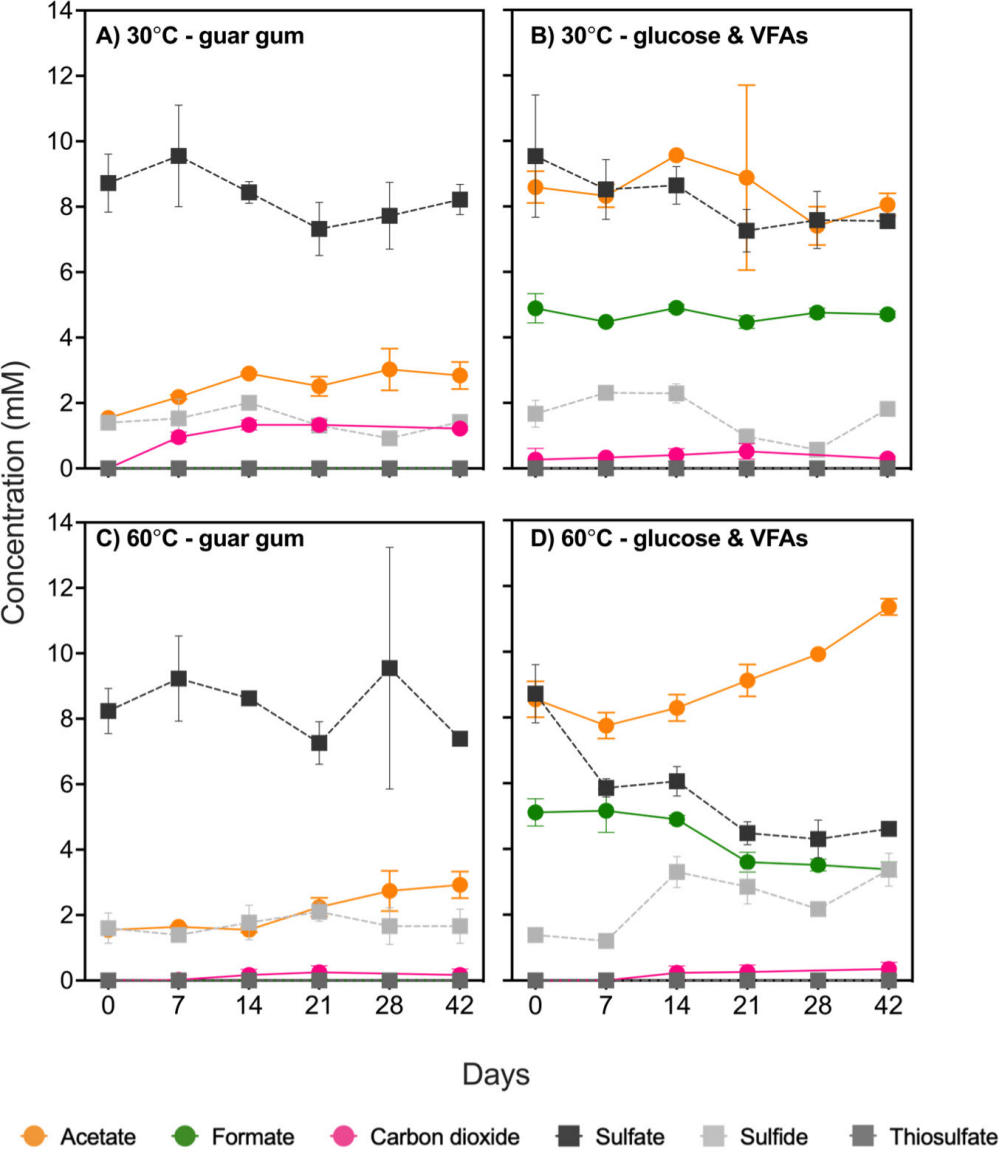
Concentrations of acetate, formate, carbon dioxide, sulfate, and sulfide throughout 42 day incubations of produced water at 30°C (A and B) and 60°C (C and D) and amended with different substrates. Error bars represent standard deviations based on triplicate incubations for each condition. Carbon compounds are represented by dots connected with solid lines, and sulfur compounds are represented by squares connected with dashed lines.

To stimulate growth in a way that mimics the presence of polymer biodegradation products, glucose and six volatile fatty acids (VFAs) were amended to the medium that was inoculated with produced water (20% vol/vol). The added 10 mM glucose was minimally consumed, decreasing by only 0.5 mM within the initial 14 days (results not shown), whereas VFAs exhibited more dynamic responses. In 60°C incubations, formate decreased between 14 and 21 days from 4.9 ± 0.1 mM to 3.6 ± 0.3 mM. Conversely, formate production was not observed in 30°C incubations with guar gum (Fig. 1A), where acetate levels increased to 2.8 ± 0.4 mM. A similar increase in acetate (up to 2.8 mM) was observed at 60°C with glucose and VFAs (Fig. 1D). Acetate was also present in the incubations that did not receive VFA amendment (Fig. 1A and C) owing to being present at 2.1 mM in the original produced water (Table S1). Carbon dioxide production was the most pronounced in 30°C guar gum incubations, reaching a final concentration of 1.2 ± 0.1 mM (Fig. 1A). Only the incubations at 60°C with glucose and VFA showed a clear decrease in sulfate concentration, which dropped from 8.7 ± 0.9 mM to 5.4 ± 0.4 mM over the 42-day incubation period, coinciding with an increase in sulfide from 1.4 ± 0.1 mM to 3.4 ± 0.5 mM (Fig. 1D). Sulfate concentrations did not change significantly in the 60°C incubation amended with guar, or in either of the 30°C incubations (Fig. 1A through C). No thiosulfate was detected throughout the microbial incubations or in the initial produced water (Table S1). No metabolic activity was observed in any of the sterile or substrate-free controls. Incubations with undiluted produced water amended with the same organic substrates to the same final concentrations showed the same trends and are presented in the supplemental materials (Fig. S1).

### Microbial community composition

**Fig 2:**
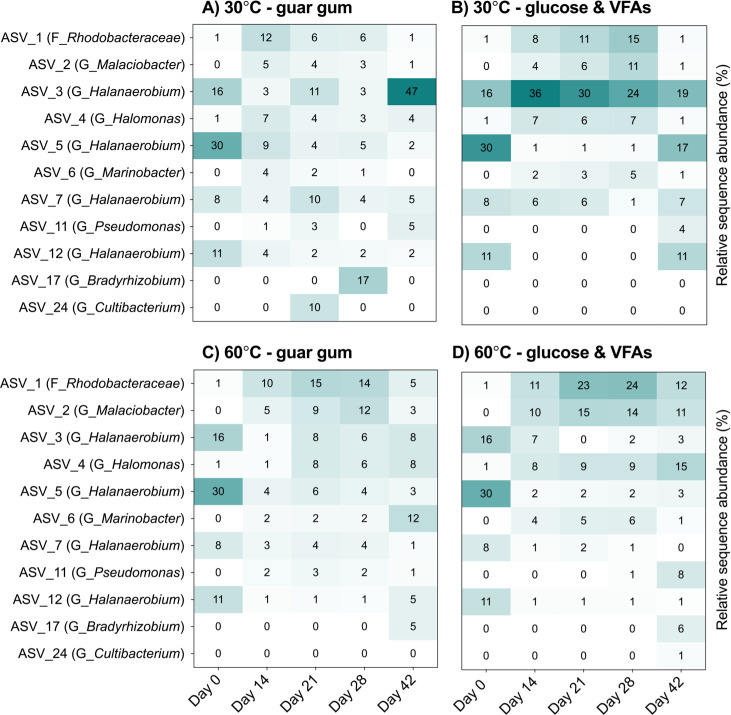
Relative sequence abundance of abundant ASVs during produced water incubations at temperatures mimicking topside storage ponds (30°C) (A and B) and subsurface oil reservoirs (60°C) (C and D). Only ASVs detected at least once at over 5% relative abundance are included. The taxonomy of each ASV is denoted in parentheses based on genus (G) or family (F) level affiliations. Values and shading indicate the average percentage of relative abundance of each ASV at a given time point, based on amplicon sequencing of triplicate incubations.

Microbial populations enriched during incubations at 30°C featured a higher relative abundance of some *Halanaerobium* amplicon sequence variants (ASVs), whereas at 60°C members of this lineage did not increase relative to initial levels detected in the produced water samples (Fig. 2). Guar gum amendment at 30°C resulted in the highest relative abundance of *Halanaerobium*, with ASV3 reaching 47% after 42 days of incubation (Fig. 2A). Similarly, *Halanaerobium* ASV7 reached 10% after 21 days in the same 30°C incubation (Fig. 2A), whereas other incubation conditions resulted in its relative abundance dropping to 1%–7% compared to being at 8% at the start of the incubation period (Fig. 2B through D). On the other hand, *Halanaerobium* ASV5 did not increase in relative abundance. Incubations with undiluted produced water showed similar shifts in microbial community composition (Fig. S2).

**Fig 3:**
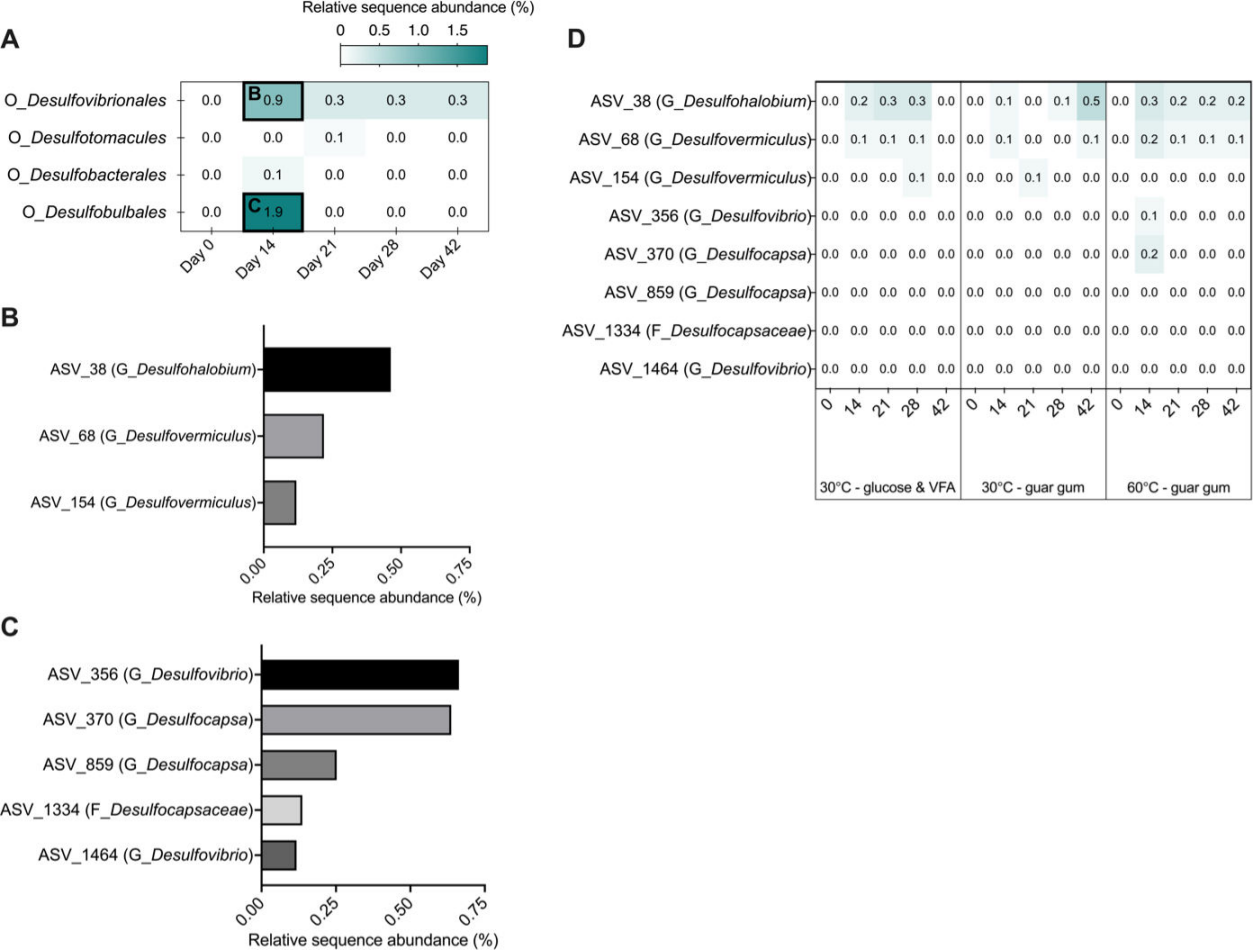
Relative sequence abundance of putative sulfate-reducing bacteria in produced water incubated at 60°C with glucose and volatile fatty acids over a 42 day period (**A**). The heatmap (**A**) shows microbial orders within the samples as the cumulative sequence abundance of ASVs affiliated with these orders throughout this incubation. Highlighted boxes are plotted in greater detail (at the individual ASV level) in histograms for *Desulfovibrionales* (**B**) and *Desulfobulbales* (**C**) corresponding to 14 days of incubation when an increase in sulfide was observed (Fig. 1D). Relative abundances of sulfate-reducing microorganisms in the three other incubation types presented in this study are also presented for comparison (**D**).

Putative sulfate-reducing bacteria were observed to increase from low levels at day 0 only in the 60°C incubations amended with glucose and VFA (Fig. 3), corresponding with sulfate reduction to sulfide (Fig. 1D). In this incubation, increases in *Desulfovibrionales* and *Desulfobulbales* were observed after 14 days of incubation, reaching 0.9% and 1.9%, respectively (Fig. 3A). Within *Desulfovibrionales*, the most prevalent genus was *Desulfohalobium* (Fig. 3B), and within *Desulfobulbales*, the genera most enriched were *Desulfovibrio* and *Desulfocapsa* (Fig. 3C). The other three incubation conditions showed very low levels (<0.5%) of putative sulfate-reducing microorganisms (Fig. 3D).

### Metagenomic analysis and quality assessment of reconstructed metagenome-assembled genomes

**Fig 4:**
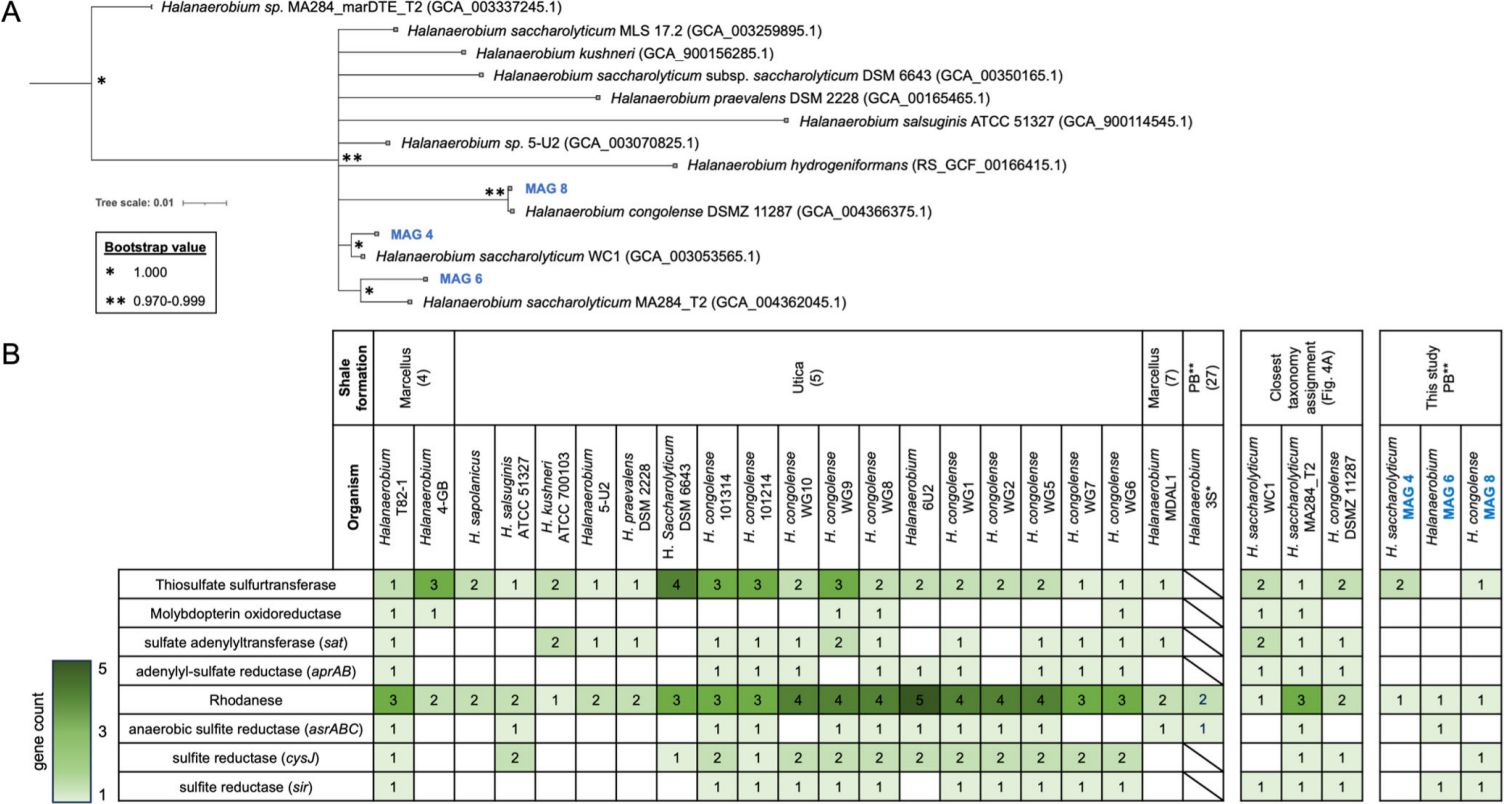
(**A**) Phylogenomic relationships among *Halanaerobiaceae* from Permian Basin produced water and other environments reconstructed from GTDK-Tk. Three *Halanaerobium* MAGs from this study (i.e., 30°C incubation with guar gum) are highlighted in bold blue font. GenBank accession numbers for genomes from other environments are indicated in parentheses. These include the type strain *Halanaerobium congolense* (18) and different *H. saccharolyticum* strains that are closely related to new MAGs uncovered in this study. The tree scale is shown at the bottom left of the figure. (**B**) Genes associated with thiosulfate metabolism/sulfur-compound disproportionation.

Metagenomic sequencing was performed on DNA extracted from the sample collected after 28 days from the 30°C guar-amended incubations and the 60°C incubations amended with glucose and VFA, based on chemical changes observed by this point (Fig. 1). A total of 25 unique medium- and high-quality metagenome-assembled genomes (MAGs) were reconstructed: 9 from 30°C incubations and 16 from 60°C incubations (Table 1; Fig. S4). MAGs belonging to the family *Halanaerobiaceae* were only retrieved from the 30°C guar gum incubations (MAG 4, MAG 6, and MAG 8; Table 1; Fig. 4). Among these, MAG 6 was classified to the genus level (*Halanaerobium*), and MAG 4 and MAG 8 were classified to the species level as *Halanaerobium saccharolyticum* and *H. congolense*, respectively. MAGs belonging to lineages known to include organisms capable of sulfate reduction were only found within the incubations at 60°C; MAG 10 and MAG 11 both belong to the order *Desulfovibrionales* and could be further classified to the order *Desulfovibrionaceae* (MAG 11) and to the species level as *Desulfohalobium retbaense* (MAG 10). Sulfate-reducing *D. retbaense* (MAG 10) had the highest replication rate among MAGs for which this feature was able to be estimated (Table 1).

**TABLE 1:** Taxonomy and predicted replication rate for 25 metagenome-assembled genomes

MAG ID	Temp (°C)	Taxonomy							Predicted replication rate	Completion (%)	Redundancy (%)
		Domain	Phylum	Class	Order	Family	Genus	Species			
**1**	30	Bacteria	Patescibacteria	Gracilibacteria	BD1-5	UBA6164	UBA6164	–^*a*^	NA^*b*^	64	0
**2**		Bacteria	Proteobacteria	Gammaproteobacteria	Pseudomonadales	*Pseudomonadaceae*	*Pseudomonas*	*Pseudomonas stutzeri*	NA	82	3
**3**		Bacteria	Proteobacteria	Alphaproteobacteria	Rhodobacterales	*Rhodobacteraceae*	*Roseovarius*	–	NA	56	3
**4**		Bacteria	Firmicutes	Halanaerobiia	Halanaerobiales	*Halanaerobiaceae*	*Halanaerobium*	*H. saccharolyticum*	NA	89	4
**5**		Bacteria	Bacteroidota	Bacteroidia	Bacteroidales	*Marinilaniliaceae*	–	–	NA	76	2
**6**		Bacteria	Firmicutes	Halanaerobiia	Halanaerobiales	*Halanaerobiaceae*	*Halanaerobium*	–	NA	93	3
**7**		Bacteria	Proteobacteria	Gammaproteobacteria	Pseudomonadales	*Halomonadaceae*	*Halomonas*	*Halomonas ventosae*	NA	99	1
**8**		Bacteria	Firmicutes	Halanaerobiia	Halanaerobiales	*Halanaerobiaceae*	*Halanaerobium*	*Halanaerobium congolense*	2.05	98	7
**9**		Bacteria	Proteobacteria	Alphaproteobacteria	Rhodobacterales	*Rhodobacteraceae*	*Roseovarius*	–	NA	97	0
**10**	60	Bacteria	Desulfobacterota	Desulfovibrionia	Desulfovibrionales	*Desulfohalobiaceae*	*Desulfohalobium*	*D. retbaense*	2.56	98	5
**11**		Bacteria	Desulfobacterota	Desulfovibrionia	Desulfovibrionales	*Desulfovibrionaceae*	–	–	NA	88	9
**12**		Bacteria	Bacteroidota	Bacteroidia	Flavobacterales	*Flavobacteraceae*	*Psychroflexus*	*Psychroflexus salarius*	NA	99	1
**13**		Bacteria	Proteobacteria	Alphaproteobacteria	Rhizobiales	*Xanthobacteraceae*	*Bradyrhizobium*	sp003020075	1.32	99	0
**14**		Bacteria	Proteobacteria	Gammaproteobacteria	Pseudomonadales	*Halomonadaceae*	*Halomonas*	*H. ventosae*	NA	93	5
**15**		Bacteria	Bacteroidota	Bacteroidia	Bacteroidales	*Proxilibacteraceae*	SKHV01	–	NA	96	3
**16**		Bacteria	Bacteroidota	Bacteroidia	Bacteroidales	UBA12170	–	–	NA	96	8
**17**		Bacteria	Campylobacterota	Campylobacteria	Campylobacterales	*Arcobacteraceae*	–	–	NA	99	4
**18**		Archaea	Halobacteriota	Methanosarcina	Methanosarcinales	*Methanosarcinaceae*	*Methanohalophilus*	–	NA	73	3
**19**		Bacteria	Thermotogota	Thermotogae	Petrotogales	–	–	–	NA	95	4
**20**		Bacteria	Patescibacteria	Gracilibacteria	Absconditabacterales	X112	–	–	NA	72	0
**21**		Bacteria	Defferibacterota	Defferibacteres	Defferibacterales	*Flexistipitaceae*	*Flexistipes*	–	NA	92	3
**22**		Bacteria	Proteobacteria	Alphaproteobacteria	Rhodobacterales	*Rhodobacteraceae*	*Roseovarius*	–	1.62	96	1
**23**		Bacteria	Firmicutes	Bacilli	Izemoplasmatales	*Izemoplasmataceae*	–	–	NA	93	3
**24**		Bacteria	Bacteroidota	Bacteroidia	Flavobacterales	*Flavobacteraceae*	*Psychroflexus*	–	NA	96	2
**25**		Bacteria	Patescibacteria	Gracilibacteria	BD1-5	UBA6164	UBA6164	–	2.5	76	1

^*a*^ – no further taxonomy is available

^*b*^ NA, not available.

### Metabolic features encoded in MAGs

#### Guar gum fermentation potential in MAGs from 30°C incubations

Guar gum biodegradation by extracellular enzymes involves β-1,4-mannanase, which catalyzes hydrolysis of the mannose backbone of the polymer, and α-1,6-galactosidase, which breaks down galactose units branching out of the mannose backbone (22). Both enzymes are encoded by *Halanaerobium* MAG 6, with *Halanaerobium* MAG 4 and MAG 8 also encoding the α-galactosidase. The β-mannanase was also found in *Marinilabillaceae* MAG 5. ABC transporters for internalization of mannose are encoded by all three *Halanaerobium* MAGs as well as by *Pseudomonas* MAG 2. The mannose phosphotransferase system (PTS) responsible for internalizing and phosphorolyzing mannose to mannose-6-phosphate was found in *Halanaerobium* MAG 8. Galactose ABC transporter genes were found in *Halanaerobium* MAG 4, MAG 6, and MAG 8, as well as in *Halomonas* MAG 7. No galactose permease genes were observed in any of the three MAGs.

**Fig 5:**
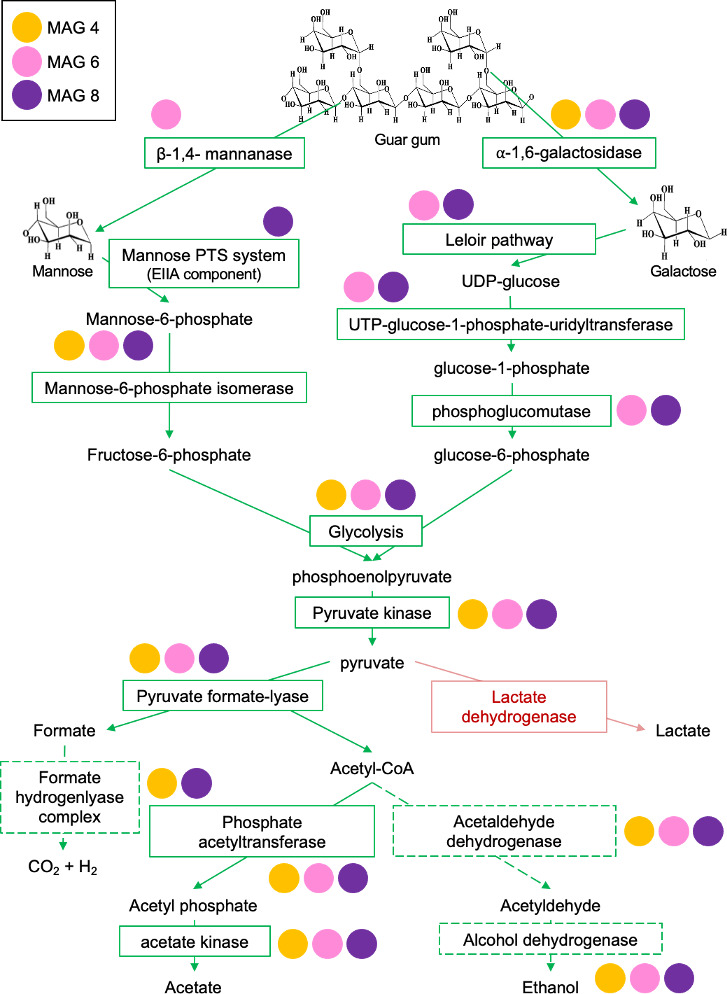
Genes present within three *Halanaerobium* genomes (Fig. 4A) encoding enzymes for catalyzing biodegradation of guar gum polymers to acetate and CO_2_ through the mixed-acid fermentation pathway (see Fig. 1A). Green arrows and boxes indicate genes and pathways detected in *Halanaerobium* MAGs 4, 6, and 8 that were enriched at 30°C in the presence of guar gum. Red arrows and boxes signify non-detection. Dashed green lines indicate partial detection of genes but not the complete pathway.

The mannose PTS (enzyme IIA [EIIA] component) is known to internalize and phosphorolyze mannose to mannose-6-phosphate (found in MAG 6), which can then be further converted to fructose-6-phosphate by the mannose-6-phosphate isomerase and introduced in the glycolysis pathway. All three *Halanaerobium* genomes encode mannose-6-phosphate isomerases and enzymes needed for glycolysis (Fig. 5). Mannose conversion to mannose-6-phosphate requires a hexokinase that was not observed in any of the MAGs retrieved from the 30°C guar gum incubations. *Halanaerobium* MAG 6 and MAG 8 have complete sets of genes for the Leloir pathway to ferment galactose to UDP-glucose. Further conversion of UDP-glucose to glucose-1-phosphate by the UTP-glucose-1-phosphate-ur(idyltransferase) and glucose-6-phosphate by phosphoglucomutase (to integrate into glycolysis) is also encoded in both genomes (Fig. 5). Mannose and galactose were below detection throughout the incubation, suggesting active internalization of sugars by *Halanaerobium*.

#### Mixed-acid fermentation potential in MAGs from 30°C incubations

Mixed-acid fermentation includes production of lactate, acetate, ethanol, CO_2_, and H_2_ via intermediate pyruvate. Genes for lactate dehydrogenase were not detected in three *Halanaerobium* MAGs or in any contigs annotated from the assemblies prior to binning, whereas genes for acetate production were found in all three *Halanaerobium* MAGs (Fig. 5). Furthermore, although complete pathways were not observed, genes for the production of CO_2_ and H_2_ are present in *Halanaerobium* MAG 4 and MAG 8. Genes encoding enzymes for ethanol production were observed in all three *Halanaerobium* MAGs. Acetate production was observed and could have been due to the conversion of pyruvate by the pyruvate oxidase gene. However, given that pyruvate oxidase was not observed in any of the three genomes, a more likely explanation is mixed-acid fermentation associated with guar gum biodegradation (Fig. 5).

#### Potential for sulfur metabolism in MAGs from 30°C and 60°C incubations

Members of the genus *Halanaerobium* have been studied for their potential to catalyze sulfidogenesis via thiosulfate reduction, yet very few genes related to thiosulfate metabolism were found in *Halanaerobium* genomes recovered from 30°C incubations with guar gum. Genes previously reported for sulfur compound disproportionation, i.e., molybdopterin oxidoreductases, SOX system genes (*SoxABCDXYZ*), sulfate adenylyltransferase, adenylylsulfate transferase subunits a and b, sulfite oxidoreductase, sulfide:quinone oxidoreductase, and sulfur oxigenase/reductase (23), were all absent from MAG 4, MAG 6, and MAG 8 despite being present in other *Halanaerobium* genomes (Fig. 4B). They were not detected in MAG 4, MAG 6, and MAG 8, which exhibited 89%–98% estimated completeness (Table 1; Fig. S4). However, thiosulfate sulfurtransferase genes were found within MAG 4 and MAG 8. A single rhodanese gene for thiosulfate conversion to elemental sulfur and sulfite was detected in all three MAGs, and genes for the further reduction of sulfite to sulfide were also detected in MAG 6 (*sir* and *asrABC*) and MAG 8 (*sir*), as shown in Fig. 5B. Genes encoding the complete pathway for dissimilatory sulfate reduction were identified in genomes assembled from the 60°C incubations classified as *Desulfohalobium* (MAG 10) and *Desulfovibrionaceae* (MAG 11). Genes for dissimilatory sulfate reduction were not detected in the metagenomes associated with incubation at 30°C.

#### Organotrophic potential of sulfate reducers and other thermophiles at 60**°C**

Genes encoding complete pathways for the oxidation of acetate to CO_2_ and acetyl-coA (acetyl-coA pathway) in MAG 10 and MAG 11 with higher replication rates (Table 1) suggest that these organisms can couple acetate oxidation to sulfate reduction at higher temperatures (60°C) in the subsurface. Highly incomplete genomic pathways for the tricarboxylic acid (TCA) cycle were retrieved from both MAG 10 and 11 with key steps missing (genes for the conversion of oxaloacetate to citrate, succinate to fumarate, fumarate to malate, and malate to oxaloacetate). Interestingly, while no genes related to hydrolysis of guar gum were found within these genomes, genes for the internalization of mannose and conversion to mannose-6-phosphate (PTS system EIIA components) were retrieved from sulfate-reducing *Desulfohalobium* MAG 10 and *Desulfovibrionaceae* MAG 11, with the latter also having hexokinase genes for converting mannose to mannose-6-phosphate. Both sulfate-reducing microorganisms have genes for the conversion of mannose-6-phosphate to fructose-6-phosphate and genes for the glycolysis pathway that are complete (MAG 10) or nearly complete (MAG 11). This indicates the potential to use mannose to complete glycolysis, coupled to sulfate reduction.

## DISCUSSION

### Fermentative metabolism under lower-temperature storage pond conditions

Members of the genus *Halanaerobium* have been extensively documented within hydraulically fractured shale oil reservoirs where they potentially catalyze fermentation (notably guar gum utilization) and/or thiosulfate metabolism (4–7, 24). Despite *Halanaerobium* being routinely recovered from shale formations with downhole temperatures up to 60°C (10), *Halanaerobium* spp. have not been cultivated above 42°C (5, 24). Consistent with these observations, *Halanaerobium* ASVs and metagenomic raw reads increased in relative abundance during incubations at 30°C with guar gum but not in 60°C incubations (Fig. 2; Table S1; Fig. 4; Fig. S4). This is corroborated by *Halanaerobium* MAG 4, MAG 6, and MAG 8 that were enriched at 30°C all having optimal growth temperatures of 39°C–40°C estimated by the Tome algorithm (25).

Previous studies have not reported on biochemical pathways for guar gum metabolism by *Halanaerobium* spp., although isolated members of this genus have been shown to biodegrade the polymer, commonly used as a gelling agent during hydraulic fracturing operations (5, 22, 26–28). Liang et al. (5) isolated a strain of *Halanaerobium* able to couple guar gum conversion to acetate with the reduction of thiosulfate to sulfide (5). In the present study, *Halanaerobium* MAGs encode mannanase and galactosidase genes for internalizing mannose and galactose derived from extracellular hydrolysis of guar gum, i.e., ABC transporters for both sugars or the mannose PTS system (Fig. 5). Once inside the cell, these sugars can be metabolized through a mixed-acid fermentation pathway (Fig. 5) to produce acetate and CO_2_, which were both observed to accumulate in the corresponding incubations (Fig. 1A).

Accordingly, fermentation of larger substrates by *Halanaerobium* (Fig. 5) may be a more plausible metabolism than thiosulfate reduction in lower-temperature topside conditions like produced water storage ponds. Fermentation appeared to be the predominant metabolism when produced water was incubated under temperatures representative of storage ponds (Fig. 1), resulting in communities dominated by *Halanaerobium* (Fig. 2). The presence of very few genes for thiosulfate metabolism in three near-complete *Halanaerobium* genomes from this Permian Basin-produced water is notable (Fig. 4B). *Halanaerobium* MAGs contain fewer genes for thiosulfate metabolism than in closely related genomes (Fig. 4A) as well as genomes analyzed in previous studies of other basins (Fig. 4B). In particular, *Halanaerobium* MAG 8 shares 97.8 average nucleotide identity with type strain *H. congolense*, an oil reservoir isolated that has been characterized as a thiosulfate reducer (18). Genes for rhodanese, which catalyzes thiosulfate disproportionation into elemental sulfur and sulfite (6, 23), were found in a single copy within *Halanaerobium* MAG 4, MAG 6, and MAG 8 compared to the multiple copies in other *Halanaerobium* strains (Fig. 4B). Genes for a thiosulfate sulfurtransferase, leading to the same intermediates as rhodanese, were found within MAG 4 and MAG 8. These enzymes alone would not result in the production of sulfide (6), whereas *asr* and *sir* genes in MAG 6 and MAG 8 enable conversion of sulfite to sulfide. Thus, while thiosulfate-based souring by those two organisms is possible in principle, levels of thiosulfate remained below detection limits within the produced water samples and during the incubation experiments (Fig. 1; Table S1). Furthermore, these *Halanaerobium* strains did not grow at temperatures representative of the oil reservoir, where thiosulfate could potentially be present (Fig. 2; Table S2).

These observations raise questions about the likelihood of thiosulfatedriven biogenic souring in oil reservoirs more generally and plausible sources of thiosulfate as a terminal electron acceptor. Oxidation of pyrite in Permian Basin formations represents one possible mechanism. However, it has been suggested that this reaction is more likely to result in sulfate generation due to the unstable nature of the thiosulfate molecule (29). Thiosulfate was not detected in the produced water samples used in this study and accordingly was not amended into incubations designed to mimic conditions in the reservoir. Had thiosulfate been included, it appears unlikely that the *Halanaerobium* strains uncovered in the 30°C incubations would have catalyzed thiosulfate reduction based on the evidence from their genomes, although it cannot be ruled out that this would have enriched different thiosulfate-reducing bacteria as other studies have demonstrated (29).

Indeed, very few studies have measured the presence of thiosulfate in produced water. Liang et al. (5) similarly found *Halanaerobium* with genes for thiosulfate metabolism but in a system with a very low thiosulfate concentration of 0.02 mM. That study conducted incubations at 40°C, whereas the formation that the samples originated from (Utica shale) reached from 55°C to 125°C (6, 10). Another strain of *Halanaerobium* able to couple thiosulfate reduction with guar metabolism was shown to do so in the presence of 10 mM thiosulfate amended to Barnett shale-produced water in which thiosulfate levels were otherwise below detection limits (5). Again, the incubation temperature was at 37°C, despite the Barnett shale formation temperatures reaching 65°C (30). Other studies of shale reservoir *Halanaerobium* found genes for the reduction of thiosulfate but did not report thiosulfate concentrations in the produced water (7, 17, 31). Based on these observations combined with results reported here, *in situ* thiosulfate reduction by *Halanaerobium* is difficult to confirm. While more work is needed to detect and measure levels of thiosulfate in these systems to understand a potential role for *Halanaerobium* in sulfide biogenesis, it is also worthwhile to consider alternate explanations for the predominance of these organisms in shale reservoir systems.

### *Halanaerobium* acting in concert with sulfate-reducing bacteria during produced water reuse

Sulfate reduction to sulfide at 60°C in incubations amended with glucose and volatile fatty acids (Fig. 1) appears to be catalyzed by thermophilic sulfate-reducing bacteria including *Desulfohalobium* MAG 10 and *Desulfovibrionaceae* MAG 11, despite amplicon sequencing suggesting these groups were present in relatively low abundance (Fig. 3) during the incubation period. At 30°C, a small drop in acetate (Fig. 1B) and a slight increase in relative abundance of putative sulfate-reducing bacteria (Fig. 3D) suggest sulfatereducing activity under these conditions should not be ruled out. Sulfate reduction corresponding to low abundances of putative sulfate-reducing microorganisms has been reported in other environmental systems (32, 33). In the 60°C incubations presented here, sulfate reduction took place during the first half of the 42-day incubation period (Fig. 1D), coinciding with the largest increase in relative sequence abundance of ASVs affiliated with known sulfate-reducing bacteria (Fig. 3). Reconstruction of *D. retbaense* (MAG 10) and a member of the *Desulfovibrionaceae* family (MAG 11) showed that in addition to complete pathways for dissimilatory sulfate reduction, these genomes contained incomplete sets of genes for the TCA cycle and all genes required for acetate oxidation via the acetyl-CoA pathway.

This understanding is consistent with *Halanaerobium* spp. being detected in saline oil fields experiencing high levels of souring caused by sulfate-reducing microorganisms (34, 35). Metagenomic sequencing points to the main role of *Halanaerobium* in this system being able to secrete extracellular enzymes to hydrolyze guar gum, thereby releasing mannose and galactose and leading to the release of other by-products (hydrogen, carbon dioxide, and acetate) for other microorganisms to utilize whether in topside storage (e.g., storage ponds) mimicked by 30°C incubations or in the subsurface mimicked by 60°C incubations. This evidence of fermentative metabolism is particularly important in the context of reinjecting produced water after a period of topside storage at lower temperature that affords *Halanaerobium* the opportunity to generate electron donors for sulfate reduction, including hexose sugars (mannose and galactose), acetate, ethanol, and hydrogen. If these compounds are introduced into the subsurface in the presence of sulfate, thermophilic sulfate-reducing microorganisms in the reservoir are presented with the opportunity to catalyze reservoir souring via dissimilatory sulfate reduction. Supporting this statement is the presence of acetate and sulfate within the sample obtained from the Permian Basin oil reservoir-produced water obtained for this study (Table S1). Also, extracellular β-1,4-mannanase secretion at storage pond temperatures for hydrolysis of guar gum would lead to increased levels of mannose (*Halanaerobium* MAG 4, MAG 6, and MAG 8), which could be directly coupled to dissimilatory sulfate reduction by *Desulfohalobium* (MAG 10) and *Desulfovibrionaceae* (MAG 11). Further studies should examine this in more detail by incubating produced water directly with by-products of guar gum biodegradation extracts as a way to provoke sulfate-reducing activity at higher temperatures.

In conclusion, produced water incubations at 30°C and 60°C demonstrated the potential for cooperative metabolic activity by *Halanaerobium* and sulfate-reducing microorganisms via produced water storage and reuse in hydraulically fractured shale formations. This highlights the importance of organotrophic metabolism by *Halanaerobium* and its potential for indirectly promoting reservoir souring by providing substrates for sulfate-reducing microorganisms. Fermentative metabolism is therefore a likely explanation for the broad prevalence of *Halanaerobium* in shale oil fields.

## MATERIALS AND METHODS

### Microbial incubations

Produced water samples from Permian Basin hydraulically fractured wells (New Mexico, USA) were used to set up microbial incubations. The medium (per 1 L) was composed of 1 g NH_4_Cl, 10 g MgCl_2_•6H_2_O, 0.1 g CaCl_2_•2H_2_O, 1 g KCl, 100 g NaCl, 0.72 g cysteine HCl, 500 µM K_2_HPO_4_/KH_2_PO_4_, 0.2% (vol/vol) NAHCO_3_, 10 mL trace element solution, and 10 mL vitamin solution (6). Microcosms were prepared by combining 20 mL saline-produced water (14.6% salinity) with 80 mL of medium in 125 mL serum bottles under anoxic conditions. Serum bottles were immediately sealed with sterile rubber stoppers, and the headspace was exchanged with N_2_:CO_2_ (90%:10%). Two different substrate types were used, either glucose (10 mM) together with a VFA mixture (acetate, butyrate, formate, lactate, propionate, and succinate, each at 5 mM) or guar gum at a concentration of 0.05% (wt/vol). Sulfate was added to all microcosms to a final concentration of 10 mM. Bottles were incubated at 30°C or 60°C. Sterile controls (medium only with substrates) and substrate-free controls (medium and produced water without substrates) were prepared and incubated in parallel. Parallel incubations with the same substrates and electron acceptors added directly to undiluted produced water were run for comparison. Subsamples from microcosms were removed using N_2_:CO_2_-flushed sterile syringes and immediately stored at −20°C until further analysis.

### Concentrations of metabolic reactants and products

Sulfate depletion was assessed by ion chromatography (Dionex ICS-5000) using an analytical column (AS23) with 8 mM Na_2_CO_3_/1 mM NaHCO_3_ eluent at a flow rate of 1 mL/min. Chromeleon software was used for visualization. Peak area calibration used Na_2_SO_4_ standard solutions. Sulfide production was measured using a spectrophotometric assay (36). Samples for sulfide analysis were collected at different time points of the experiment and mixed with zinc acetate (20% [vol/vol]) to limit sulfide volatility (37). Samples were kept frozen until analyzed.

Volatile fatty acid levels were measured using high-performance liquid chromatography in an Ultimate 3000 RSLC system with a 5 mM H_2_SO_4_ mobile phase at a flow rate of 0.6 mL/min and a temperature of 60°C using a Bio-Rad Aminex HPX-87H column. Carbon dioxide production was measured using gas chromatography with a flame ionization detector by injecting headspace gas into an Agilent 7890B gas chromatograph equipped with a Hayesep N packing column (stainless steel tubing, 0.5 m length × 1/8 inch outer diameter × 2 mm internal diameter, mesh size 80/100). Helium was used as a carrier gas (flow rate 21 mL/min). The oven temperature was set to 105°C, and carbon dioxide was detected by a thermal conductivity detector set to 200°C.

### Genomic DNA extraction

Five-milliliter aliquots were removed from microcosms at days 0, 7, 14, 21, and 28 and then frozen until genomic DNA was extracted using the DNeasy PowerSoil extraction kit (Qiagen) following the manufacturer’s instructions. Resulting DNA concentrations were measured by fluorimetry (Qubit, Qiagen). DNA was used for both 16S rRNA gene amplicon sequencing and shotgun metagenomic sequencing, as described below.

### 16S rRNA gene amplicon sequencing and sequence analysis

The V4 hypervariable region of the 16S rRNA gene was amplified using primers 515F (GTGYCAGCMGCCGCGGTAA) and 806R (GGACTACNVGGGTWTCTAAT). PCR reactions with a total volume of 25 µL included 2× KAPA HiFi Hot Start Ready Mix (KAPA Biosystems), a final concentration of 0.1 mM for each primer, and 7.5 µL of DNA sample. PCR included an initial denaturation at 95°C for 5 min followed by 30 cycles of denaturation at 95°C for 30 seconds, annealing at 55°C for 45 seconds, and extension at 72°C for 1 min. This was followed by a final extension for 5 min at 72°C. PCR products followed post-PCR. Triplicate PCR products were pooled prior to cleanup and indexing. Indexed amplicon samples were sequenced using Illumina’s v3 600-cycle (paired-end) reagent kit on an in-house Illumina MiSeq benchtop sequencer (38) after all DNA extraction blanks and PCR reagent blanks were confirmed negative for amplification. Quality-controlled reads were merged and subsequently dereplicated to construct an ASV table. Relative abundance calculations were based on the number of unrarefied reads per sample. Analysis and visualization were done using R and GraphPad Prism (39, 40).

### Metagenomic sequencing

Genomic DNA extracted from produced water incubations after 28 days of incubation was sent to the Centre for Health Genomics and Informatics (University of Calgary, Calgary, Canada) for library preparation using sonication (Covaris) and sequencing using a NovaSeq platform (Illumina). Adapters were removed; the last 151 bp of the last read was trimmed; contaminants (PhiX control V3 library, Illumina) were filtered; and lowquality ends were clipped off with BBDuk (41). Assembly was done with Megahit using a co-assembly for replicates of a given sample, and reads were assembled using Bowtie2 (42, 43). DasTool allowed for binning of the contigs while combining outputs from Concoct, Maxbin, and Metabat (44–46). Quality assessment of the bins was performed with CheckM “lineage_wf” (v.1.1.2) with taxonomy assigned using GTDB-Tk “classify_wf” (v.1.5.0) (47, 48). Only bins with <10% contamination and >70% completeness were retained for annotation and further study. DRAM (v.1.2) was used to annotate dereplicated MAGs for functional potential (49, 50). PhyloFlash allowed for taxonomic identification from the metagenomic reads (51). Estimations of temperature optima for MAGs used Tome (25). iRep allowed for estimation of replication rates on MAGs (52). Genomes outside the minimal requirements (under 2% contamination, over 75% complete) to analyze with iRep were excluded from the analyses.

### Comparative analyses of other halanaerobium genomes

Genomes from other studies (5–7, 31) were downloaded from the National Center for Biotechnology Information (https://www.ncbi.nlm.nih.gov/) or JGI (https://genome.jgi.doe.gov/portal/) as an assembly FASTA file and annotated with DRAM for comparison of thiosulfate-related metabolism.

### Phylogeny

Phylogenomic trees were constructed using CheckM (v.1.1.3) and included 2,052 finished genomes and 3,604 draft genomes from the Integrated Microbial Genomes database (45). Genes were aligned using GAMMA and WAG models. Nodes were interpreted as bootstrap values, and the tree was constructed using Dendroscope for visualization (53).

## Data Availability

The data discussed in this paper are available online under GenBank BioProject number PRJNA972118.

## SUPPLEMENTAL MATERIAL

Supplemental material aem.02253-24-s0001.docx Figures S1 to S4; Tables S1 and S2.

ASM does not own the copyrights to Supplemental Material that may be linked to, or accessed through, an article. The authors have granted ASM a non-exclusive, world-wide license to publish the Supplemental Material files. Please contact the corresponding author directly for reuse.
