# Supplementary material for: Polymer biodegradation by Halanaerobium promotes reservoir souring during hydraulic fracturing
Source: Appl Environ Microbiol. 2025 Apr 9;91(5):e02253-24. doi: 10.1128/aem.02253-24 (PMC12093946; doi:10.1128/aem.02253-24)
Supplement: Supplemental material — Figures S1 to S4; Tables S1 and S2. [file aem.02253-24-s0001.docx]

**
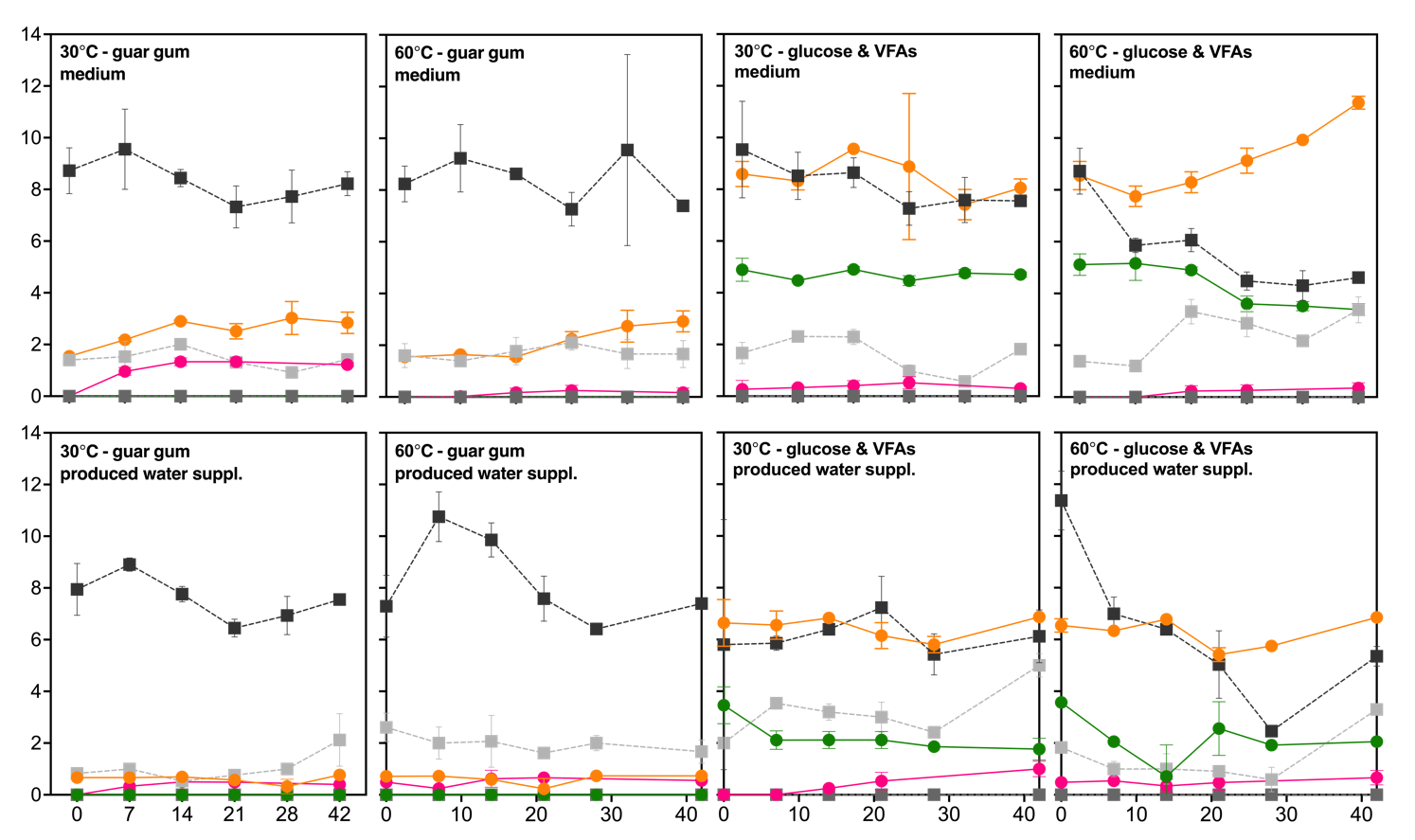
**

**Figure S1**: Concentrations of acetate, formate, carbon dioxide, sulfate and sulfide throughout 42-day incubations of produced water at 30°C and 60°C amended with different substrates. The top row shows medium inoculated with produced water, and the bottom row shows produced water amended directly with the substrates indicated. Error bars represent standard deviation based on triplicate incubations for each condition. Carbon compounds are represented by dots connected with solid lines and sulfur compounds are represented by squares connected with dashed lines.

**
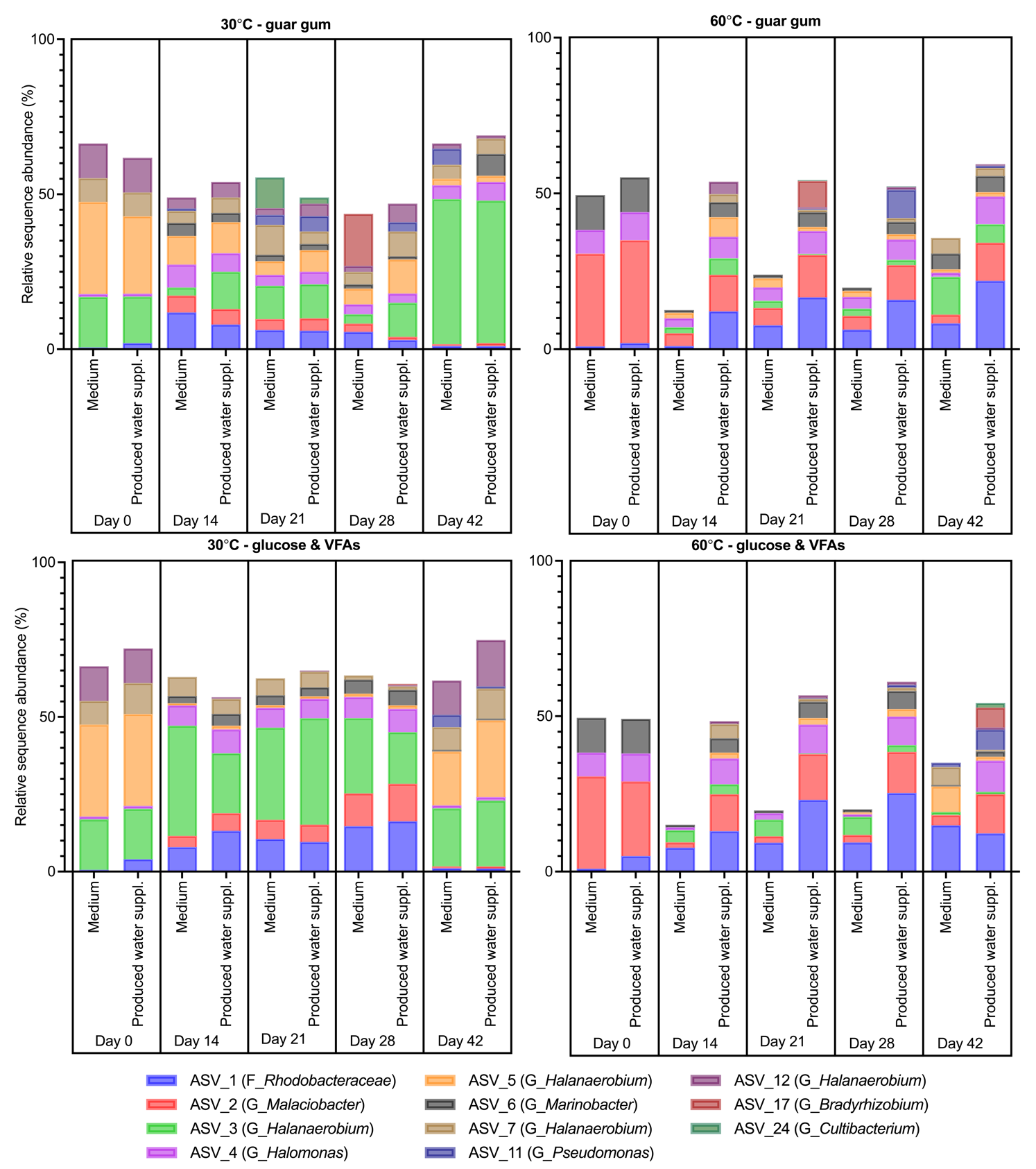
**

**Figure S2:** Relative sequence abundance of prominent ASVs in 30 and 60°C incubations of medium inoculated with produced water (“Medium”) and produced water supplemented directly with the substrates indicated above each of the panels (“Produced water suppl.”). The two temperatures were selected to mimick topsides storage ponds (30°C) and subsurface oil reservoirs (60°C). Only ASVs detected at least once at >5% are included. Taxonomy of each ASV is denoted in parentheses based on genus (G) or family (F) level affiliations.

**
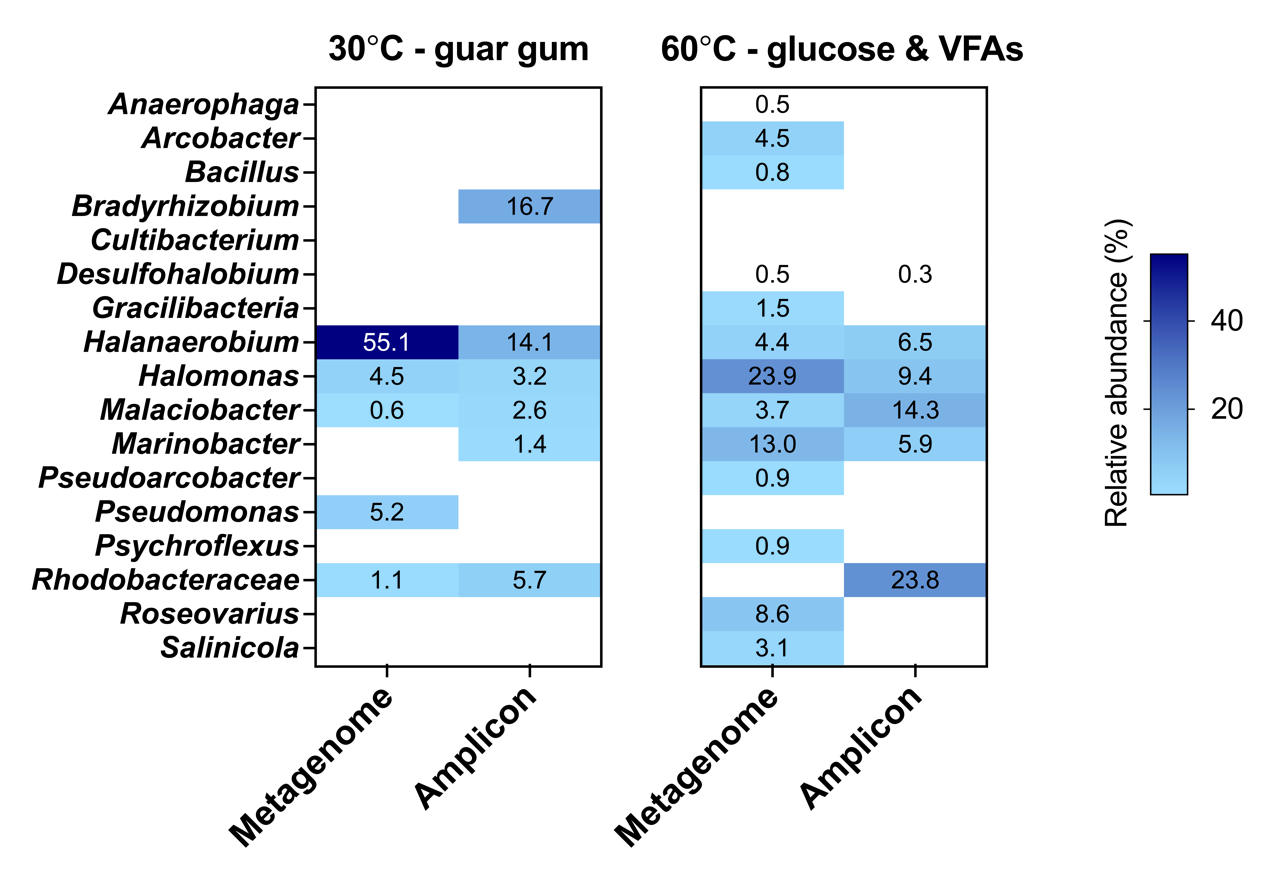
**

**Figure S3:** Microbial community composition based on metagenome reads at 28 days (labeled “metagenome”) of incubation from samples mimicking topsides temperature (30°C; supplemented with guar gum) and subsurface oil reservoirs temperature (60°C; supplemented with glucose and VFA) compared to amplicon sequencing data (labeled “amplicon”, Figure 2). Only ASVs over 0.5% relative abundance in either group of enrichments are included.


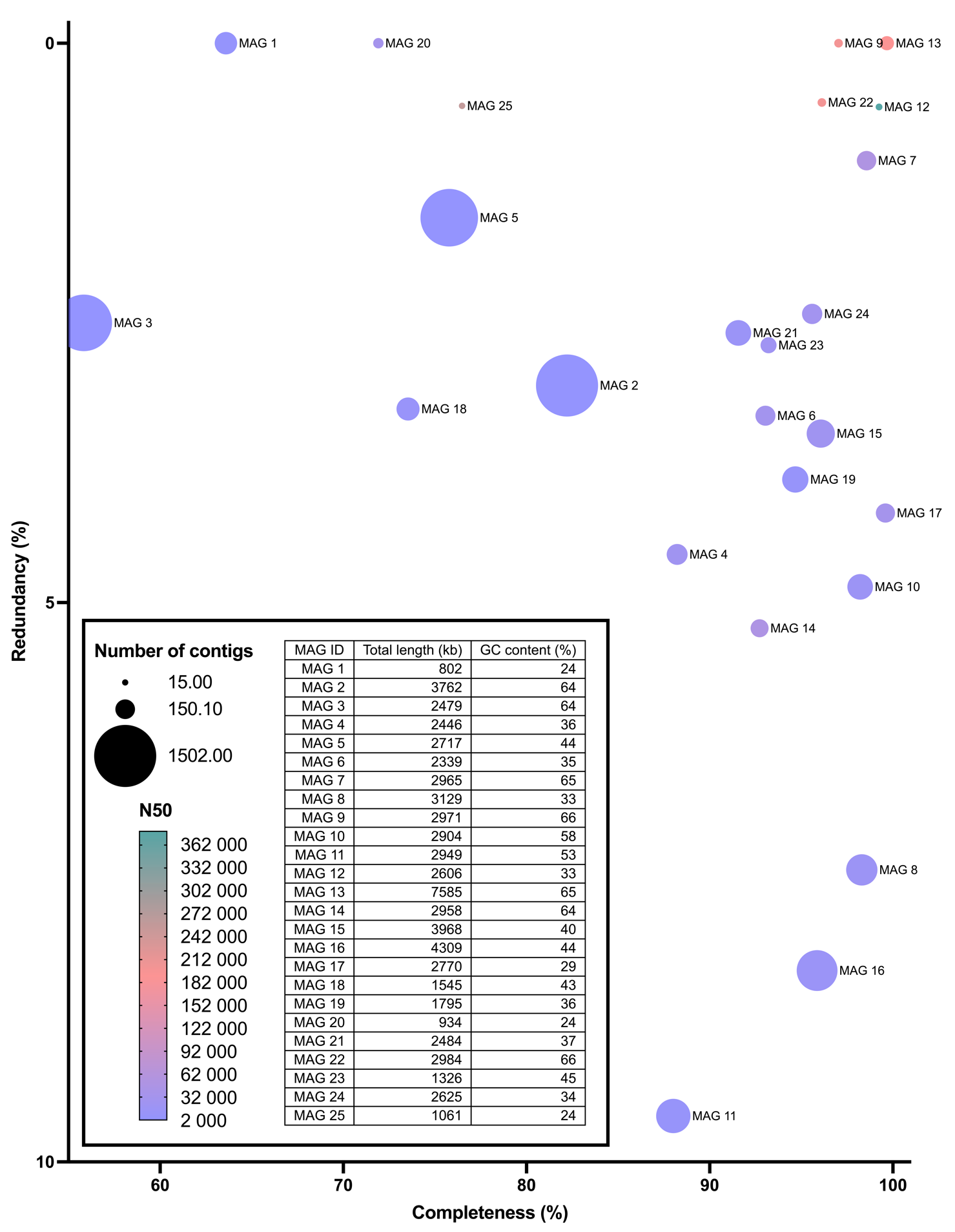


**Figure S4:** Quality of 25 bins retrieved from the Permian Basin produced water enrichments after 42 days of incubations.

13 **Table S1:** Concentration of various compounds measured within the Permian Basin produced 14 water used for microbial enrichments in this study.

| **Compound** | **Concentration (mM)** |
| --- | --- |
| Sulfate | 4.1 |
| Thiosulfate | Below detection limits |
| Acetate | 2.1 |
| Butyrate | Below detection limits |
| Formate | ND |
| Lactate | Below detection limits |
| Propionate | 0.2 |
| Succinate | ND |

15

16

17 **Table S2:** Optimal growth temperature computationally predicted using Tome for three different 18 Halanaerobium MAGs found within the 30°C enrichments with guar gum.

| **MAG ID** | **Predicted optimal growth temperature (**°**C)** |
| --- | --- |
| 4 | 39 |
| 6 | 39 |
| 8 | 40 |

19

20

21

22
